# Socioeconomic risk factors and age-related macular degeneration in the UK Biobank study

**DOI:** 10.1136/bmjophth-2020-000585

**Published:** 2021-02-23

**Authors:** Jennifer Lai Yee Yip, Zaynah Muthy, Tunde Peto, Andrew Lotery, Paul J Foster, Praveen Patel, Additional UK Biobank Eye and Vision Consortium Membership, Tariq Aslam

**Affiliations:** 1 Clinical Research Department, London School of Hygiene & Tropical Medicine, London, UK; 2 Institute of Ophthalmology, UCL, London, UK; 3 NIHR Moorfields Biomedical Research Centre, London, Greater London, UK; 4 Centre for Public Health, Blackwell's Queen's University Belfast, Belfast, UK; 5 Faculty of Medicine, University of Southampton, Southampton, Hampshire, UK

**Keywords:** retina, public health, epidemiology, degeneration, epidemiology

## Abstract

**Objective:**

There is contrasting evidence on the relationship between socioeconomic status (SES) and age-related macular degeneration (AMD), the most common cause of visual impairment (VI) in developed countries. This study examines the relationship between SES, cardiovascular risk factors and self-reported AMD.

**Methods and analysis:**

Over 500000 people participated in the UK Biobank study from 2006 to 2019, with sociodemographic data and clinical measurements collected using standardised procedures. Visual acuity was measured in 117907 participants with VI defined as LogMAR ≤0.3. We used logistic regression to examine the cross-sectional associations between SES and self-reported AMD.

**Results:**

Self-reported AMD was available for 133339 participants aged 50 and older. People reporting AMD had higher academic qualifications, lower income, were unable to work due to disability, have higher BMI, diabetes and vascular heart disease after adjusting for age and sex. In a multivariable analysis, higher income was protective of AMD and economic inactivity due to disability increased the odds of AMD (2.02, 95% CI 1.13 to 3.61). Both associations were independent of cardiovascular factors, but was no longer significant after adjusting for VI.

**Conclusions:**

The association between education, employment and household income with AMD was independent of cardiovascular risk factors.

**Key messages**

**What is already known about this subject?**

- Age-related macular degeneration (AMD) is a common cause of blindness globally. Lower socioeconomic status (SES) is associated with higher levels of visual impairment (VI), but the association with AMD is unclear.

**What are the new findings?**

- People with higher income were less likely to report AMD; also, that economic inactivity due to disability was associated with AMD.

**How might these results change the focus of research or clinical practice?**

- These results should focus greater public health efforts to address eye health inequalities, targeting preventive messages for people at greater risk of AMD.

## Introduction

Age-related macular degeneration (AMD) is the leading cause of low vision and blindness in high-income countries.^1 2^ Early forms of the disease may be asymptomatic and are characterised by drusen deposits adjacent to the retinal pigment epithelium but AMD can progress to more advanced forms of the condition leading to loss of central vision. Advanced forms of AMD are characterised by thinning or loss of the outer retina, retinal pigment epithelium and choriocapillaris (geographic atrophy, an advanced form of ‘dry’ AMD) or through the development of choroidal neovascularisation (‘wet’ form of advanced AMD). These forms of advanced AMD damage central vision with significant impact on an individual’s quality of life and daily function. AMD causes approximately 5% of global blindness, with an estimated 71000 new cases of late AMD per year in the UK.^3^ This is expected to increase due to the ageing population as older age is the strongest risk factor for AMD and over 600000 people in the UK are estimated to suffer from AMD by 2020.^3^

The WHO Commission on Social Determinants of Health have highlighted the importance of socioeconomic risk factors in the aetiology of chronic disease and disability.^4^ Addressing health inequalities is a key driver in national and international health policy. People living in poorer areas, from less affluent backgrounds, have a higher risk of morbidity and mortality. There is substantial evidence that lower socioeconomic status (SES) is associated with visual impairment (VI),^5–7^ higher prevalence and incidence of eye disease,^8–10^ and ocular risk factors.^11–14^ There is limited information on the association between individual SES and AMD. The Beaver Dam study showed that higher SES, as measured by years of education and type of employment, but not income, was directly and independently associated with higher 5-year incidence of AMD.^15^ Lower educational attainment, but not lower income, was also independently associated with higher AMD prevalence in studies from Italy, Singapore and Korea.^16–18^ There is also evidence that people with AMD are more likely to live in more deprived areas.^19^ These studies indicate that lower SES may increase the risk of AMD.

Cardiovascular risk factors are associated with higher prevalence and incidence of AMD.^20–25^ Pooled analysis from the Beaver Dam, the Blue Mountains and Rotterdam Eye Studies showed that current smokers were more than twice as likely to develop late AMD than people who had never smoked.^26^ Other identified vascular associations with AMD include cardiovascular disease,^23 27^ stroke,^28^ hypertension^23^ and inflammatory markers such as C- reactive protein (CRP).^29 30^ Smoking and cardiovascular disease are more prevalent in people with lower SES and living in more deprived areas. These common risk factors could indicate common pathological mechanisms, and cardiovascular risk factors may mediate the association between SES and AMD.

In this study, we examined the relationship between socioeconomic factors, area deprivation, cardiovascular risk factors and self-reported AMD using the UK Biobank resource.

## Methods

The UK Biobank (UKBB) resource is a large-scale collection of health information that includes data collected at 22 study assessment centres, from 502 656 participants in the UK registered with the National Health Service, aged 40–69 years in 2006–2010. The overarching aim of UK Biobank is to improve the prevention, diagnosis and treatment of a wide range of serious illnesses including cancer, heart diseases, stroke, diabetes, arthritis, osteoporosis, eye disorders, depression and dementia. The study protocol (http://www.ukbiobank.ac.uk/resources/) and protocols for individual tests (http://biobank.ctsu.ox.ac.uk/crystal/docs.cgi) are available online. Informed consent was obtained from all participants. Patients and the public were not involved in the design, or conduct, or reporting or dissemination plans in our research.

All participants were examined using standardised procedures across sites. A touch screen self-administered questionnaire was used to collect information on their general health, SES and lifestyle factors. Ethnicity was recorded by the participant as white, Chinese, Asian, black or mixed/other. Smoking status, both past and current, was determined through questionnaire. Diabetes status was determined as those who answered yes to ‘Has a doctor ever told you that you have diabetes?’ Socioeconomic information included education (qualifications attained, grouped into three categories: higher academic/professional (college or university, A levels or above, professional qualifications), lower academic/vocational (O levels or equivalent, CSEs or equivalence, NVQ, HND or equivalent) and other (none of the above)), employment (in paid employment, retired, looking after home/family, unable to work because of sickness/disability, unemployed, unpaid/voluntary work, student, or none of the above), and annual household income (less than £18 000, 18000–30 999, 31000–51 999, 52000–100000, greater than 100 000). The Townsend Deprivation Index is a marker of material deprivation, where higher scores represent higher levels of deprivation. The index is based on census data from the participants’ postcode, and aggregated from measures of unemployment, car ownership, home ownership and household overcrowding.

Glaucoma and macular degeneration statuses were determined as those who selected ‘glaucoma’ or ‘macular degeneration’ from a list of eye disorders to the question ‘Has a doctor told you that you have any of the following problems with your eyes?’ Summary data of the whole cohort were obtained from the online data showcase (http://www.ukbiobank.ac.uk/data-showcase/).

In 2009, an additional ophthalmic examination was appended to the main protocol and we obtained LogMAR visual acuity on 117 907 participants. Presenting visual acuity was measured, with the participant wearing optical correction as prescribed. Visual acuity was measured using a bespoke UK Biobank testing software on a display screen with standard illumination with the room lights turned off. More information is available in the UK Biobank Visual Acuity testing manual on the Biobank website. Visual impairment was defined in this study as VA worse than LogMAR 0.3 in the better eye (Snellen equivalent of cannot see 6/12 in better eye).

### Physical measurements

Blood pressure and heart rate were measured using the Omron HEM-70151T digital blood pressure monitor. Two measurements of each were taken and the mean was used in subsequent analysis. Weight was measured with the Tanita BV-418 MA body composition analyser. Height was measured using a Seca 202 height measure. Body mass index (BMI) was calculated as weight/height^2^. Waist circumference at the level of the umbilicus was measured using a Wessex non-stretchable sprung tape measure. Baseline visual acuity was measured using a computerised semiautomated LogMAR system at 3 m, with best available correction.

### Statistical analysis

Analyses were conducted on data from participants with complete data for all variables of interest. χ^2^ tests and t-tests were used to examine the relationship between categorical variables and quantitative variables, respectively.

Logistic regression was used to explore the univariable associations with AMD, and multivariable regression was used to estimate adjusted OR for AMD. Confounders were identified a priori (age, sex and smoking) or detected through observed associations with both the exposure (socioeconomic variables) and outcome of interest. Indicators were used to relax underlying assumptions on the distributions of categorical variables. We first explored multivariable associations adjusting for age and sex to gain a better understanding of the underlying associations independent of age and sex. A stepwise forward fitting model was built using the Wald (binary or quantitative variable) or likelihood ratio test (multilevel categorical variable) to determine if each new variable included in the model was independently associated with the outcome, adjusting for previously included variables. All variables were then checked for significance against the final proposed model. A separate analysis was conducted on the participants with visual acuity measurement due to the smaller sample size.

## Results

Of 502 656 participants in the baseline Biobank study, we excluded those who were under 50 years old (117 906), those who did not respond to the self-reported eye diseases question (247 411) or reported null answers (3985), and a further 15 with missing data, leaving 133 339 participants with responses to the self-reported AMD questions.

In this study of 133 339 people, 3634 people aged 55 and older reported a diagnosis of AMD, with study prevalence of 2.72% (95% CI 2.63 to 2.81). The mean age was 61.01 (95% CI 69.98 to 61.04) with median age of 61.66 (IQR 56.75–65.50). There were 72 906 women (54.7%), and 60 433 men (45.3%) who responded to the question on whether they had received a diagnosis of AMD. A large proportion of the study participants were white (93.4%), with blacks and South Asians forming 2.1% and 2.5%, respectively, 1.1% reported ‘other’, 0.6% mixed and 0.4% East Asian.

Table 1 shows the characteristics of the study population by age groups. There were higher proportions of women in younger age groups compared with men. There were also higher proportions of ethnic minorities, current smokers, people in paid employment, higher levels of educational attainment and with higher household incomes in the younger age groups. Older people were more likely to have higher BMI, systolic BP, but lower diastolic BP and pulse rate, and live in more affluent areas. The majority of people in the 65–70 year age group were retired, as expected, with income in the lower brackets, and more likely to be previous smokers.

**Table 1:** Distribution of exposure and confounder variables by age groups

Variables	Age groups				*P value
	50–54	55–59	60–64	65–70	
Female gender (% (n))	56.92 (13 655)	56.32 (16 404)	54.85 (23 623)	51.73 (19 224)	<0.001
Ethnicity (% (n))					<0.001
White	88.85 (21 229)	92.17 (26 750)	95.21 (40 825)	95.22 (35 204)	
Black	4.19 (1001)	2.32 (672)	1.33 (569)	1.47 (544)	
East Asian	0.54 (129)	0.55 (161)	0.25 (106)	0.23 (86)	
South Asian	3.63 (868)	2.96 (859)	1.98 (848)	1.98 (732)	
Any Mixed	1.02 (244)	0.64 (186)	0.42 (178)	0.39 (146)	
Other	1.76 (421)	1.36 (394)	0.82 (353)	0.71 (261)	
BMI (mean (CI))	27.58 (27.51–27.65)	27.61 (27.55–27.67)	27.67 (27.62–27.71)	27.63 (27.59–27.68)	=0.114
Smoking status (% (n))					<0.001
Never	59.37 (14 207)	55.09 (15 996)	51.63 (22 147)	49.34 (18 241)	
Previous	28.39 (6795)	34.55 (10 031)	39.89 (17 109)	43.69 (16 152)	
Current	12.24 (2929)	10.36 (3009)	8.48 (3638)	6.97 (2576)	
Systolic BP (mean (CI))	133.91 (133.70–134.13)	137.41 (137.19–137.60)	141.58 (141.41–141.75)	145.31 (145.12–145.50)	<0.001
Diastolic BP (mean (CI))	82.63 (82.50–82.76)	82.70 (82.59–82.82)	82.41 (82.31–82.50)	81.65 (81.55–81.75)	<0.001
Diabetes (% (n))	5.49 (1313)	6.86 (1992)	7.66 (3291)	9.61 (3559)	<0.001
Vascular/Heart disease (% (n))	24.47 (5858)	30.97 (9002)	37.97 (16 328)	46.61 (17 281)	<0.001
Waist/Hip ratio (mean(CI))	0.87 (0.87–0.87)	0.88 (0.87–0.88)	0.88 (0.88–0.88)	0.89 (0.89–0.89)	<0.001
Pulse rate (mean(CI))	69.95 (68.80–69.07)	68.93 (68.80–69.06)	69.11 (69.00–69.22)	69.10 (69.98–69.22)	=0.032
Qualifications (% (n))					<0.001
None of the above	9.7 (2300)	13.4 (3855)	20.91 (8882)	29.66 (10 840)	
Lower academic/vocational	35.2 (8344)	31.9 (9171)	31.1 (13 206)	29.0 (10 580)	
Higher academic/professional	55.1 (13 064)	54.7 (15 733)	48.0 (20 394)	41.4 (15 125)	
Employment (% (n))					<0.001
None of the above	0.72 (171)	0.84 (243)	0.61 (262)	0.43 (160)	
In paid employment	80.9 (19 298)	68.1 (19 738)	36.92 (15 841)	13.2 (4891)	
Retired	3.83 (914)	15.76 (4568)	56.24 (24 132)	85.29 (31 615)	
Looking after home and/or family	4.02 (960)	4.14 (1201)	1.15 (492)	0.47 (492)	
Unable to work because of sickness or disability	6.05 (1444)	7.14 (2069)	3.31 (1419)	0.2 (75)	
Unemployed	3.56 (848)	3.06 (888)	1.24 (531)	0.06 (22)	
Doing unpaid or voluntary work	0.61 (146)	0.75 (217)	0.47 (203)	0.33 (122)	
Full or part-time student	0.3 (72)	0.21 (60)	0.07 (28)	0.02 (8)	
Household income (% (n))					<0.001
Less than 18 000	15.81 (3348)	19.27 (4867)	28.74 (10 290)	39.04 (11 591)	
18 000 to 30 999	19.76 (4184)	23.29 (5882)	30.21 (10 815)	33.88 (10 059)	
31 000 to 51 999	28.26 (5984)	27.29 (6892)	23.61 (8452)	17.96 (5333)	
52 000 to 100 000	27.72 (5869)	23.67 (5976)	13.88 (4969)	7.39 (2194)	
Greater than 100 000	8.44 (1787)	6.47 (1635)	3.57 (1278)	1.74 (516)	
Townsend Deprivation Index (mean (CI))	−0.78 (−0.82 to −0.74)	−1.07 (−1.10 to −1.03)	−1.32 (−1.34 to −1.29)	−1.35 (−1.38 to −1.32)	<0.001

*P value from Wilcoxon rank-sum test for continuous variables and Ψ^2^ test for categorical variables.

BMI, body mass index; BP, blood pressure.

Table 2 shows the associations between sociodemographic variables, cardiovascular variables and AMD. There were higher proportions of people with AMD in older age groups, and who were male. There were greater proportions of people who were white, East Asian, mixed and other who reported AMD. People who reported AMD were also more likely to have VI, to be previous smokers, higher systolic but lower diastolic BP, to report diabetes, vascular, heart disease and have higher pulse rate. People with AMD were also more likely to be retire, or unable to work because of sickness or disability, and have lower levels of household income. There were higher proportions of people who reported AMD with VI.

**Table 2:** Distribution of exposure and confounder variables by AMD status

Variables	Outcome status		P value*
	No AMD (n=129 705)	AMD (n=3634)	
Age groups (% (n))			<0.001
50–54	18.26 (23 684)	8.45 (307)	
55–59	22.03 (28 580)	14.97 (544)	
60–64	32.3 (41 890)	32.33 (1175)	
65–70	27.41 (35 551)	44.25 (1608)	
Female gender (% (n))	54.51 (70 704)	60.59 (2202)	<0.001
Ethnicity (% (n))			=0.004
White	93.38 (120 600)	94.22 (3408)	
Black	2.11 (2731)	1.52 (55)	
East Asian	0.36 (462)	0.55 (20)	
South Asian	2.51 (3240)	1.85 (67)	
Any mixed	0.56 (729)	0.69 (25)	
Other	1.07 (1387)	1.16 (42)	
Visual impairment (% (n))			<0.001
Visual impairment (≥0.3)	3.62 (3138)	8.67 (103)	
BMI (mean(CI))	27.63 (27.60–27.65)	27.75 (27.59–27.92)	=0.118
Smoking status (% (n))			=0.013
Never	53.19 (68 730)	51.42 (1861)	
Previous	37.64 (48 639)	40.01 (1448)	
Current	9.16 (11 842)	8.57 (310)	
Systolic BP (mean(CI))	140.29 (140.18–140.39)	141.74 (141.12–142.35)	<0.001
Diastolic BP (mean(CI))	82.31 (82.26–82.37)	81.89 (81.53–82.19)	=0.007
Diabetes (%(n))	7.6 (9,825)	9.11(330)	=0.001
Vascular/Heart disease (% (n))	36.24 (46 919)	42.74 (1550)	<0.001
Waist/Hip ratio (mean (CI))	0.88 (0.88–0.88)	0.88 (0.87–0.88)	=0.002
Pulse rate (mean (CI))	69.02 (68.95–69.08)	69.86 (69.48–70.24)	<0.001
Qualifications (% (n))			=0.06
None of the above	19.64(25 127)	21.00 (750)	
Lower academic/vocational	31.45 (40 230)	29.99 (1071)	
Higher academic/professional	48.91 (62 566)	49.01 (1750)	
Employment (% (n))			<0.001
None of the above	0.63 (817)	0.52 (19)	
In paid employment	45.39 (58 642)	31.1 (1126)	
Retired	45.68 (59 013)	61.2 (2216)	
Looking after home and/or family	2.15 (2781)	1.27 (46)	
Unable to work because of sickness or disability	3.75 (4846)	4.45 (161)	
Unemployed	1.75 (2256)	0.91 (33)	
Doing unpaid or voluntary work	0.52 (671)	0.47 (17)	
Full or part-time student	0.13 (165)	0.08 (3)	
Household income (% (n))			<0.001
Less than 18 000	26.7 (29 085)	33.64 (1011)	
18 000 to 30 999	27.6 (29 085)	29.18 (877)	
31 000 to 51 999	23.86 (25 988)	22.4 (673)	
52 000 to 100 000	17.13 (18 656)	11.71 (352)	
Greater than 100 000	4.7 (5124)	3.06(92)	
Townsend Deprivation Index (mean (CI))	−1.17 (−1.19 to −1.15)	−1.30 (−1.40 to −1.20)	<0.001

*P value from Wilcoxon rank-sum test for continuous variables and Ψ^2^ test for categorical variables. All totals may not add up to 133 339 due to missing data.

AMD, age-related macular degeneration; BMI, body mass index; BP, blood pressure.

The age and sex-adjusted associations with AMD are shown in table 3 as the associations shown in table 2 indicated that the direction of effect between SES variables were aligned with that for age and sex. After adjusting for age and sex, East Asians were more likely to report AMD. People with lower academic or vocational qualification, or reported ‘none of the above’ had lower odds of reporting AMD after adjusting for age and sex. An association was also observed with those unable to work because of sickness or disability. People living in less deprived areas, with higher levels of income, those reporting diabetes, vascular/heart disease (VHD) and higher BMI also had higher odds of AMD. There was also a strong direct association between AMD and VI (OR=2.34, 95% CI 1.90 to 2.87, p<0.01).

**Table 3:** Age and sex-adjusted associations with age-related macular degeneration

	AMD		P value*
	OR	95% CI	
Ethnicity (% (n))			**0.01**
White	Ref		
Black	0.85	0.65 to 1.11	0.23
East Asian	1.80	1.14 to 2.82	**0.01**
South Asian	0.84	0.65 to 1.07	0.15
Any mixed	1.42	0.95 to 2.12	0.09
Other	1.24	0.91 to 1.70	0.16
Visual impairment	2.34	1.90 to 2.87	**<0.01**
Qualifications			**<0.01**
None of the above	Ref		
Lower academic/vocational	1.08	0.98 to 1.19	0.12
Higher academic/professional	1.16	1.07 to 1.27	**<0.01**
Employment			**<0.01**
None of the above	Ref		
In paid employment	1.00	0.63 to 0.80	1.00
Retired	1.14	0.72 to 1.81	0.57
Looking after home and/or family	0.82	0.48 to 1.40	0.47
Unable to work because of sickness or disability	1.87	1.15 to 3.02	**0.01**
Unemployed	0.90	0.51 to 1.60	0.73
Doing unpaid or voluntary work	1.06	0.54 to 2.05	0.87
Full or part-time student	1.06	0.31 to 3.62	0.93
Townsend index			**<0.01**
Quintile 1 (most deprived)	Ref		
Quintile 2	0.95	0.86 to 1.06	0.35
Quintile 3	0.93	0.84 to 1.03	0.18
Quintile 4	0.84	0.76 to 0.94	**<0.01**
Quintile 5	0.94	0.85 to 1.05	0.28
Household income			**<0.01**
Less than 18 000	Ref		
1 8 000 to 30 999	0.90	0.82 to 0.99	0.02
31 000 to 51 999	0.94	0.85 to 1.04	0.25
5 000 to 1 00 000	0.80	**0.70 to 0.90**	**<0.01**
Greater than 100 000	0.79	0.63 to 0.98	0.03
Smoking			
Never	Ref		
Ever	1.06	0.99 to 1.13	0.12
BMI (per 1 kg/m^2^ increase)	1.01	1.00 to 1.01	0.04
Systolic BP (per 1 mm Hg increase)	1.00	1.00 to 1.00	0.58
Diastolic BP (per 1 mm Hg increase)	1.00	1.00 to 1.00	0.62
Diabetes (Yes)	1.17	1.04 to 1.32	**<0.01**
Vascular/Heart disease (Yes)	1.17	1.10 to 1.26	**<0.01**
Total	**133 198†**		

*P value from Wald test.

†Missing covariables were excluded from analysis.

AMD, age-related macular degeneration; BMI, body mass index; BP, blood pressure; ref, reference level.

The multivariable associations with AMD in the final model are shown in table 4. Older age, being male, East Asian and having VHD were all independently associated with increased odds of AMD. Any qualifications, employment and household income were socioeconomic indicators independently associated with AMD, whereas Townsend index was not a significant risk factor for AMD after adjusting for other SES variables, and therefore not included in the final model. Those reporting any qualifications had increased odds of AMD compared with those stating ‘none of the above’. There was evidence of a linear trend in the association between household income and odds of AMD, with those with higher income having lower odds of AMD, with the most affluent households earning greater than 100 000K per year with a 24% reduced odds of AMD (OR 0.76, 95%CI 0.61 to 0.96, p=0.02). People who were unable to work because of sickness and disability were twice as likely to report AMD (OR 2.20, 95% CI 1.13 to 3.61, p=0.02). We included VI in the final model to determine whether VI played a role in the relationship between SES and AMD with complete data on 73 748 participants. After adjusting for VI, only educational attainment, but not employment or income, was associated with AMD.

**Table 4:** Multivariable associations with age-related macular degeneration

	AMD (Model 1)			AMD (Model 2)		
	OR	95% CI	P value*	OR	95% CI	P value*
Age (per year increase)	1.09	1.08 to 1.10	**<0.01**	1.10	1.08 to 1.12	<0.01
Sex (Female)	0.74	0.69 to 0.80	**<0.01**	0.75	0.66 to 0.86	<0.01
Visual impairment	–	–	–	2.46	1.95 to 3.10	<0.01
Ethnicity (% (n))			**<0.01†**			0.14†
White	Ref			Ref		
Black	0.84	0.61 to 1.16	0.29	1.19	0.78 to 1.84	0.42
East Asian	1.72	1.02 to 2.90	**0.04**	**1.29**	0.48 to 3.48	0.62
South Asian	0.90	0.68 to 1.16	0.44	1.34	0.93 to 1.92	0.11
Any mixed	1.60	1.04 to 2.47	0.03	1.66	0.82 to 3.38	0.16
Other	1.48	1.06 to 1.23	0.02	1.75	1.07 to 2.86	0.03
Qualifications			**<0.01†**			**<0.01†**
None of the above	Ref			Ref		
Lower academic/vocational	1.16	1.03 to 1.29	**0.01**	1.46	1.20 to 1.78	**<0.01**
Higher academic/professional	1.30	1.16 to 1.45	**<0.01**	1.20	0.98 to 1.47	0.08
Employment			**<0.01†**			0.23†
None of the above	Ref			Ref		
In paid employment	1.06	0.61 to 1.85	0.84	0.58	0.28 to 1.18	0.13
Retired	1.15	0.66 to 2.01	0.61	0.62	0.30 to 1.26	0.19
Looking after home and/or family	0.99	0.52 to 1.88	0.98	0.49	0.19 to 1.23	0.13
Unable to work because of sickness or disability	2.02	1.13 to 3.61	**0.02**	0.99	0.44 to 2.21	0.97
Unemployed	0.88	0.44 to 1.73	0.70	0.57	0.23 to 1.45	0.24
Doing unpaid or voluntary work	1.09	0.50 to 2.37	0.84	0.67	0.22 to 2.07	0.49
Full or part-time student	0.89	0.20 to 4.00	0.88	–	–	–
Household income			**<0.01^§^**			0.42†
Less than 18 000	Ref			Ref		
18 000 to 30 999	0.90	0.81 to 0.99	0.03	0.88	0.74 to 1.05	0.16
31 000 to 51 999	0.94	0.84 to 1.05	0.27	1.00	0.83 to 1.21	1.00
52 000 to 100 000	0.80	**0.69 to 0.92**	**<0.01**	**0.88**	0.70 to 1.11	0.28
Greater than 100 000	0.76	0.61 to 0.96	0.02	0.81	0.56 to 1.18	0.28
Smoking						
Never	Ref					
Ever	1.02	0.05 to 1.10	0.55	1.01	0.89 to 1.15	0.88
Vascular/Heart disease (Yes)	1.14	1.05 to 1.23	**<0.01**	**1.13**	0.99 to 1.29	0.07
Total	110 589			**73 748‡**		

Model 2 includes visual impairment, in addition to covariables included in Model 1.

*P value from Wald test.

†From likelihood ratio test.

‡There were missing variables for VI (see the Methods section).

AMD, age-related macular degeneration; ref, reference level.

## Discussion

AMD is an important public health problem worldwide, with incidence and prevalence increasing due to an ageing population,^3^ and no treatment for a majority of cases.^25^ The adverse impact of AMD extends beyond loss of visual function with increased risk of poor mental and physical health from depression and falls.^31–33^

In this large community-based cohort study, we have shown that people living in the most affluent households have 24% reduced odds of AMD compared with those living in the poorest households, after adjusting for age, sex, education, employment and smoking. Our findings support the study from Zhang *et al*, examining data from the National Health and Nutrition Examination Surveys (NHANES III and NHANES 2005–2008). They found higher age and sex-standardised prevalence of AMD in people with lowest levels of income compared with those with the highest income level in NHANES III (17.9% vs 11.5% p=0.03), but not in NHANES 2005–2008 (10.4% vs 6.8%, p=0.06).^8^

Although we detected an association with qualifications, we found that people with higher academic qualifications or having professional qualifications had increased risk of AMD. This finding appears to contrast with other studies which report an increased risk of AMD with lower lever levels of educational attainment^16^ and several studies that have failed to detect an association between AMD and education, including results from India^34^ and the USA.^8 35^ In this study, we used those who stated ‘none of the above’ as the reference group. This group had lower household income, were predominantly in the oldest age groups (41.9% vs 23.5% in higher academic and 25.6% in lower academic qualifications) and more likely to be retired. This could indicate a selection bias with a relatively healthy older group, who have lower levels of educational attainment and live in poorer households, but were sufficiently healthy and interested to participate in a research study. Another explanation could be reporting bias, given the self-reported nature of the outcome, as people with higher levels of education may be more aware of the presence of early disease, possibly identified in routine eye checks.

People with the lowest income were older and more likely to report AMD. After adjusting for age, sex, ethnicity, and other SES and clinical factors, we showed that people with higher incomes were less likely to report AMD, which suggests that this relationship is independent of other SES factors. Although other studies have not reported an independent effect of income once education was accounted for,^16–18^ this study had greater power to detect smaller effects through a larger sample size. Similar to other SES factor assessed, the relationship of income and AMD was no longer significant after adjusting for VI. This suggests that it is impaired vision, and possibly disability as discussed next, that is key to the relationship between low income and AMD.

We found that people who were unable to work due to sickness or disability had twice the odds of reporting AMD. There could be a bidirectional relationship in this association, those with cardiovascular risk factors may have pre-existing disease and disability in addition to increased risk of AMD, and people with AMD can experience and register disability which limits their ability to work and gain income. AMD is the most common cause of registered sight loss in the UK. We cannot determine direction of association with this cross-sectional study. Nonetheless, that AMD is associated with twice the risk of economic inactivity due to sickness and disability is an important consideration for health and social care, and reinforces the need for greater focus on treatment and prevention of this condition.

This study also showed that the association between individual SES indicators and AMD was independent of Townsend index, age, sex and smoking. The Townsend index is based on four questions and has been superseded by the index of multiple deprivation (IMD) in the UK, which aggregates 38 markers of deprivation. A study from Norfolk, UK, has shown that those living in more affluent areas, as measured by IMD, had lower risk of AMD, independent of individual SES including education and social class.^19^ It is likely that Townsend is a closer proxy to individual SES compared with IMD, given that IMD includes additional measures of area crime, and the living environment; and the effect of area deprivation as measured by Townsend may have been mediated by the markers of individual SES in this study.

We included VI as an additional covariable in the final model to determine whether this would affect the detected relationships between SES indicators and AMD. Higher income no longer held a protective effect on AMD after adjusting for VI. Similarly, employment status, in particular, disability was also no longer associated with AMD after adjusting for VI. This suggests that it is visual function that determines the relationship between income, disability and AMD. As this is a cross-sectional analysis, we cannot determine direction of association, and it is possible that people with AMD and poorer vision and more likely to report disability and have lower levels of household income. This contrasts to our previous analysis examining the association between SES and self-reported glaucoma, where higher income conferred lower odds of reported glaucoma, independent of VI.^36^ These findings could indicate different mechanisms through which social determinant may impact on different eye diseases.

Smoking is a strong risk factor for AMD,^26^ and people with lower SES are more likely to smoke.^37^ However, in our study population, despite people living in poorer households being more likely to be current smokers, we did not detect an association between cigarette smoking and AMD, likely due to low power in a healthy population with low prevalence of current smokers (9.15%, 95% CI 8.99% to 9.30%). Therefore, although our results indicate that the association between individual SES and AMD is independent of smoking, we cannot rule out this mechanism in other populations. The relationship between SES and AMD was also independent of diabetes and vascular/heart disease. Other potential downstream mechanisms of SES include lifestyle risk factors such as diet, as people in more affluent social groups have diets with higher levels of serum carotenoids,^38^ and lutein and zeaxanthin are principal components of macular pigment with important roles in visual function.^39^

The UK Biobank is a large prospective population-based cohort, and the large sample size and extensive information allow for examination of a wide range of exposures. Here, we have focused on socioeconomic determinants, commonly considered as the ‘causes of causes’ in the aetiology of disease.^40^ Although large in size, Biobank UK had a response rate of around 5.5%, which will limit its generalisability. However, provided there is sufficient variation in the distribution of exposures and outcomes in a large sample, the detected associations can be generalisable, although the point estimates may differ.^41^ We used self-reported measures for ascertainment of both exposure and outcome, which can lead to measurement bias. Studies have shown that self-reported measures have high levels of accuracy (>80%) in well-defined chronic conditions such as ischaemic heart disease, diabetes and hypertension, but lower for stroke.^42–44^ However, non-differential measurement bias can remain and result in inaccurate estimates. The accuracy of self-reported estimates for AMD, a primarily asymptomatic disease, is poorer, with likely underestimation of prevalence.^45–47^ The impact of this limitation may have been reduced power of the study to detect an association. Our sample included people aged under 70, which would exclude people at highest risk and with more severe disease. This would have led to lower estimated frequency of AMD, and possibly underestimated the association between SES and AMD, as older people were also more likely to be retired and report lower household incomes. Although we did detect an association with the key SES variables, the effect of these limitations cannot be quantified and may have resulted in an underestimate or overestimate of the main relationship examined.

This is the largest analysis of the relationship between SES and AMD to date, which showed higher levels of educational attainment and unemployment due to disability were associated with higher levels of AMD, and higher household income was associated with lower prevalence of AMD. These associations with SES were independent of cardiovascular risk factors.
